# ***The Power of Plagues, 2***^***nd***^ ***Edition.*** Sherman IW. Washington, DC: ASM Press, 2017

**DOI:** 10.4269/ajtmh.18-0942

**Published:** 2019-02

**Authors:** Marlene Zuk

**Affiliations:** Department of Ecology, Evolution and Behavior; University of Minnesota; Minneapolis, MN; E-mail: mzuk@umn.edu

The world is awash in books about disease, and most of these fall into one of two categories. First are the ones with chipper, upbeat (and virtually always incorrect) advice about how “one simple trick,” whether via diet, exercise, supplements, or another practice, can cure all ills and save the reader from the dire medical establishment and Big Pharma. Alternatively, we have what I call “Ebola is coming and we are all going to die” books, which invoke grim statistics about the state of our readiness to deal with the new epidemic that is about to spiral out of control.

Mercifully, Irwin Sherman’s new edition of *The Power of Plagues* follows neither of these overworked tropes. In the spirit of full disclosure, I should point out that the author and I were colleagues at the University of California, Riverside, for many years, and that he wrote the first edition of the book as an accompaniment to a nonmajors course that he, and later I, taught there. The book, and the course, was designed not only to show students how important human diseases have been in the course of history, but also to teach basic principles of biology such as the differences between bacteria and viruses or the nature of the immune system. Understanding T cells and lymph nodes for their own sake is not nearly as compelling as understanding them because they provide an insight into how vaccination works or why acquired immune deficiency syndrome (AIDS) is so difficult to treat. The book is thus a window into biology motivated by interest in pathogens and how they have changed—and been changed by—human behavior.

*The Power of Plagues* takes a very loosely historical approach to a host of important diseases, starting with the way that ancient diseases such as tuberculosis, malaria, or measles were facilitated by humans settling down into sedentary communities. It does not attempt to be comprehensive, and readers may find that their “favorite” parasite is left out, but each disease is a fulcrum for making a larger point. Thus, cholera serves to illustrate how microbes were eventually recognized as the cause of many diseases, with a lesson about the classic experiments by Francesco Redi, Lazzaro Spallanzani, and Louis Pasteur along the way (Incidentally, I often ask classes to predict the outcome of the famous swan neck flasks with broth experiments that Pasteur designed; it is surprising how difficult a task they find this, underscoring how nonintuitive the results of such experiments can be.) Smallpox highlights the social context of vaccination, and Sherman notes that compulsory vaccination has a long history. The Germans required that all citizens, and especially members of the army, be vaccinated against smallpox by 1869, whereas no such rule existed in France, with the result that in the Franco–Prussian War, smallpox killed nearly eight times more French than German soldiers.

Every chapter is filled with people who were instrumental in describing, treating, or curing a disease, and although Sherman mentions the usual suspects—Ross, Selman, and Jenner—he also highlights some of the lesser known figures in the history of disease, such as William MacCallum and Eurgene Opie from Johns Hopkins, who used avian malaria to elucidate the life cycle of the blood parasites in humans. He also notes some controversies surrounding major achievements in medical history: why, for example, did Salk, Koprowski, and Sabin not share in the Nobel Prize for developing the polio vaccine? According to Sherman, in Salk’s case, it is because “Salk had broken two of the commandments of scientific research. Thou shalt give credit to others. Thou shalt not discuss one’s work in newspapers and magazines.” It is interesting to reflect on the relevance of the latter statement in this age of social media—if Twitter were available, Salk would have been a formidable tweeter, I suspect, and it likely would have done him more good than harm.

Other controversies are almost consoling to read about because they remind us that acrimony over scientific credit and accomplishments is nothing new, particularly when the conflicts are overlain with nationalism. The Italian scientist Giovanni Battista Grassi demonstrated that mosquitoes transmit human malaria and expected that he—and his country—would be recognized for this groundbreaking accomplishment, but was challenged by the British physician Ronald Ross, whose name is more commonly associated with the discovery. The two squabbled about priority, literally for decades, and it was Ross, but not Grassi, who received the Nobel Prize in 1902.

In addition to chapters centered on particular diseases and their associated concepts (cholera and public sanitation, AIDS and the immune system), *The Power of Plagues* considers the ways in which anesthesia, antiseptics, and antibiotics transformed medicine. The history of the first is particularly colorful, and Sherman explains that until reliable anesthetics were available, surgeons prided themselves on the speed with which they could accomplish their task. One such surgeon, Robert Liston, is said to have “amputated a leg in two and a half minutes, but the patient died shortly thereafter from gangrene (as was often the case in those days): in addition, he amputated the fingers of his young assistant, who also died later from gangrene, and slashed the coattails of a distinguished surgical spectator, who was so terrified that the knife had pierced his vital organs that he dropped dead in fright. So ended the only operation in history with 300% mortality!” We would all do well to remember those days, as did a Thanksgiving essay by A. J. Jacobs on gratitude from the *New York Times*, which mused, “Whenever I start to mythologize the past, I repeat a three-word mantra I made up: ‘Surgery without anesthesia.’ (https://www.nytimes.com/2018/11/17/opinion/sunday/thanksgiving-gratitude-thanks.html?action=click&module=Opinion&pgtype=Homepage).

The chapter on syphilis gives a particularly compelling lesson on how public perception of a disease influences the way it is managed; the notion that those who contracted a sexually transmitted infection were to blame for their sins led to misinformation about syphilis being transferred on pens, drinking cups, or toilet seats. Immigrants and sex workers were also targeted as centers of infection, with an anti-sexually-transmitted infection effort during World War I resulting in the quarantine of thousands of women living and working near military training sites.

This new edition includes more images, most in color, than the first edition, which was published in 2006. They range from historical illustrations, such as the one showing the first operation conducted with the patient anesthetized with ether, to the obligatory gory photographs of people afflicted with river blindness or Hansen’s disease. The new edition also provides a useful “coda” at the end of each chapter, offering a succinct summary of the major events in history affected by each disease and how the disease illustrates scientific advances.

The book is written for a general audience, and works well as an undergraduate text, although it probably does not have enough biological detail for a class focusing on the biology, rather than the social and historical context, of disease. It makes for good coffee-table browsing, although perhaps not for the overly squeamish (see aforementioned images), and serves as a valuable reminder that plagues, perhaps even more than the poor, will always be with us.

